# Fever: Its Pathology and Treatment

**Published:** 1891-11

**Authors:** 


FEVER: ITS PATHOLOGY AND TREATMENT. By Antipyretics. Being an Essay which was awarded the Boylston Prize of Harvard University, July, 1890. By Hobart Amory Hare, M. D., B. Sc., Clinical Professor of Diseases of Children and Demonstrator of Therapeutics in the University of Pennsylvania. Philadelphia and London: F. A. Davis, Publisher, 1891. No. 10 in "The Physician's and Student's Ready Reference Series." Price, $1.25, net.

This is a beautiful little book gotten up by the distinguished young clinician, Dr. Hare, in which he gives the experimental and clinical evidence of

Antipyrin, Antifebrin, Thallin, Phenacetine, and Salicylic-acid. It must have cost the Doctor a great deal of time and labor to gather together such a mass of evidence from the writings of German, English, French, and American experimentalists regarding these new remedies. The treatise will no doubt find a ready sale amongst our allopathic friends, because in a nutshell they can here find true reports from their best men regarding these chemicals.

Dr. Hare is a very young man, but has already achieved distinction by his several learned essays, and has been made editor of *The Medical News* and a Professor in Jefferson Medical College, of Philadelphia. W. S.

